# Safety analysis in patients with autoimmune disease receiving allogeneic mesenchymal stem cells infusion: a long-term retrospective study

**DOI:** 10.1186/s13287-018-1053-4

**Published:** 2018-11-14

**Authors:** Jun Liang, Huayong Zhang, Wei Kong, Wei Deng, Dandan Wang, Xuebing Feng, Cheng Zhao, Bingzhu Hua, Hong Wang, Lingyun Sun

**Affiliations:** Department of Rheumatology and Immunology, the Affiliated Drum Tower Hospital of Nanjing University Medical School, 321 Zhongshan Road, Nanjing, Jiangsu CN 210008 People’s Republic of China

## Abstract

**Objective:**

The aim of this study was to evaluate the safety of mesenchymal stem cell infusion in patients with autoimmune diseases.

**Methods:**

A total of 404 patients with autoimmune diseases who received mesenchymal stem cell infusion between 2007 and 2016 were included in this study. Adverse events in these patients were collected, mainly including infections and malignancies. Sources of information included hospitalization records and data from outpatient visits and each follow-up.

**Results:**

The mean follow-up period of all patients was 43.4 ± 25.9 months (range 1–109). Majority of stem cells were from the umbilical cord. The most common indications for mesenchymal stem cell infusion were systemic lupus erythematosus, Sjögren’s syndrome, and systemic sclerosis. The median age at infusion was 38.7 ± 15.7 years. The 5-year and 8-year survival rates were 90.4% and 88.9%, respectively. Median follow-up of survivors was 45.1 ± 25.7 months. The incidence rate of infections was 29.5% (119/404), and that of serious infections was 12.9% (52/404). Five patients (1.2%) experienced malignancies. Deaths occurred in 45 patients, and transplantation-related mortality was 0.2%. The most common causes of deaths in our study were disease relapse and complications associated with the underlying disease.

**Conclusion:**

Autoimmune disease is an emerging indication for mesenchymal stem cell infusion. Our data shows that mesenchymal stem cell infusion is a safe therapy for patients with autoimmune diseases. The incidences of adverse events, whether infections or malignancies, are acceptable in these patients.

**Trial registration:**

ClinaicalTrials.gov, NCT00698191. Registered 17 June 2008—Retrospectively registered

## Introduction

Mesenchymal stem cell infusion (MSCI) has been described as a promising cell-based therapy, mainly because the cells display potent immunomodulatory and immunoprivilege properties and remarkable reparative capacities [1–7]. Recently, MSCI has been applied to treat a variety of autoimmune diseases (AID), including systemic lupus eryhtematosus (SLE), Sjögren’s syndrome (SS), scleroderma (SSc), polymyositis and dermatomyositis (PM/DM), vasculitis, and rheumatoid arthritis (RA) [8–10]. Many clinical researchers have reported the efficacy of MSCI in these diseases, but they may underestimate the safety profile of MSCI. One of the important reasons is attributed to relative short-term follow-up and small sample size limit in these clinical trials.

Mesenchymal stem cells (MSCs) have been found to inhibit proliferation and function of many allogeneic immune cells including T cell, B cell, and dendritic cell, mostly by paracrine mechanisms through a wide range of soluble factors [11, 12]. However, these immune cells play key roles in host defense against viral infections and immune surveillance against cancer. Furthermore, MSCs can inhibit protein production and gene expression of tumor necrosis factor-α (TNF-α), which plays a critical role in protection against many infections. So, some unfavorable consequences might arise when applying MSCs as cell therapy in AID patients. In theory, the risk of infection and carcinogenesis might increase associated with dysfunction of specific effector cells or decreased TNF-α in AID patients receiving cell infusion. Other possible adverse events (AEs) may be caused by cell infusion itself, such as pulmonary cytolytic, venous thromboembolism, fever, and graft versus host disease, which are often reported in systematic reviews and meta-analyses about hematopoietic stem cell transplantation (HSCT) [13–15].

In this context, there is an essential need to conduct a study, with relatively larger sample size and longer follow-up time, aiming to evaluate the safety profile after MSCI. In order to do that, all patients who received MSCI in our center were enrolled into this study. Safety was compared between MSCI and HSCT about the incidence of reported adverse events, mainly infections and malignancies.

## Material and methods

### Type of study and population

We performed a retrospective study in a cohort of 404 patients with different autoimmune diseases undergoing MSCI at the Affiliated Drum Tower Hospital of Nanjing University Medical School during the period of 2007 to 2016. This study was conducted in accordance with the Declaration of Helsinki and approved by the hospital ethical committee (Nos: 2006006, 2008017, 2009004). This trial was registered in ClinicalTrials.gov (identifiers: NCT00698191, NCT00953485, NCT00962923, NCT01741857). Clinical data, mainly about infusion-related AEs, were collected retrospectively from each follow-up, and the medical records of inpatient and outpatient. For this analysis, all the staffs in our center were required to document any new sign or symptom of each enrolled patient, any hospitalization or mortality and their reasons, and any worsening of the baseline comorbid conditions, irrespective a causal relationship with the infusion, at any time point during the whole follow-up period.

### MSCs isolating and culturing

The sources of MSCs involved in this study were the bone marrow (BM) and umbilical cord (UC). BM-MSCs and UC-MSCs were harvested as described previously with slight modification [8, 16]. Donors of BM aged between 18 and 40 years were selected from the members of the patients’ healthy families without HLA matching. Bone marrow cells were collected by gradient centrifugation and seeded at a density of 1 × 10^6^ cells/cm^2^ in growth medium containing Dulbecco’s modified Eagle medium, low glucose (DMEM-LG; Gibco) and 10% fetal bovine serum (FBS) (HyClone). The non-adherent cells were removed after 72 h, and the medium was changed twice weekly thereafter. Fresh UC was obtained from healthy mothers after normal deliveries and were rinsed twice in PBS consisting of penicillin and streptomycin to remove the cord blood. Then, the washed cords were cut into 1-mm^2^ pieces and floated in low-glucose DMEM containing FBS. The pieces of cord were subsequently incubated at 37 °C in a humidified atmosphere consisting of 5% CO_2_ in air. As the cells grew to 80% confluence, adherent cells were replated at a density of 1 × 10^4^ cells/cm^2^ in growth medium for expansion. After two passages, the cells were harvested. The infusion of MSCs to the patients should finish within 8 h after cell acquisition.

### Infusion procedures

All the enrolled patients had been diagnosed to have one of AID, such as SLE, SS, SSc, PM/DM, RA, mixed connective tissue disease, autoimmune liver disease, and primary vasculitis. They had not responded to at least one course of conventional treatment and still had high disease activity. Exclusion criteria included the presence of active untreated infectious disease caused by any viruses and bacteria, or the presence of any types of malignancies. All the patients received allogeneic MSCI for treatment of the underlying autoimmune diseases. Before infusion, informed consents were obtained from the patients and their families. Each patient received the same infusion procedure, which was not altered with disease severity or disease course. MSCs at a dose of 1 × 10^6^ cells per kg of body were suspended in 100 ml saline and slowly infused by a heparinized syringe through the cubital vein of the arm over 30 min. These patients were discharged at least after 24 h of observation.

### Outcome measures

The primary endpoint of this study was to evaluate the frequency of AEs, including hyperacute adverse events (haAEs), acute adverse events (aAEs), organ system-related adverse events, infections, malignancies, and deaths. AEs were analyzed according to the Common Terminology Criteria for Adverse Events version 4.0 (CTCAE v4.0) [17] and were divided into five grades: grade 1, mild: asymptomatic or mild symptoms, clinical or diagnostic observations only, intervention not indicated; grade 2, moderate: local or noninvasive intervention indicated; grade 3, severe or medically significant but not immediately life-threatening, hospitalization or prolongation of hospitalization indicated; grade 4, life-threatening consequences, urgent intervention indicated; and grade 5, death related to AEs. Grades 3–5 were considered serious AEs (sAEs). Hyperacute adverse events were defined as occurring during and immediately after the infusion, the latter was set as the first day (D1) after MSCI. Acute adverse events were defined as occurring from the second day (D2) to the first month after infusion.

The incidences of infections and malignancies occurring after infusion were also calculated. Any definition of infections or cancers should have positive results from either hospital data or confirmation of the patients and their families. Time-to-infection (TTI) was defined as the time interval between MSCI and the first positive infection. Time-to-cancer (TTC) was defined as the time interval between MSCI and the first positive cancer.

Overall survival rates and all the deaths in infused patients were analyzed. Time-to-death (TTD) was defined as the time interval between MSCI and patient death. Assessment of transplantation-related mortality (TRM), a very important term in HSCT and defined as any death within 100 days after infusion of MSCs in the absence of relapse or progression of underlying autoimmune diseases, was also included among the objectives of this study.

### Statistical analysis

Data were presented as means ± SEM. Frequencies and percentages were used for categorical variables, and comparisons were tested by Pearson chi-square test or Fisher’s exact test, as appropriate. Survival curves were estimated using the Kaplan-Meier method and were tested between groups using the log-rank test. Statistical analysis was performed with SPSS16.0 software or GraphPad Prism 5. A level of two-sided *p* value < 0.05 was considered to indicate statistical significance.

## Results

### Characteristics of the study population at the time of enrollment

In total, 404 patients underwent the allogeneic MSCI from 2007 to 2016 at the Affiliated Drum Tower Hospital of Nanjing University Medical School. Patient characteristics are summarized in Table 1. The median age of patients submitted to MSCI was 38.7 ± 15.7 years (range, 7–79), and the mean follow-up of all patients was 43.4 ± 25.9 months (range 1–109). There were 60 males (14.9%) and 344 females (85.2%). The main indications for MSCI were SLE (*n* = 178; 44.1%), SS (*n* = 72; 17.8%), SSc (*n* = 39; 9.7%), and RA (*n* = 32; 7.9%) and PM/DM (*n* = 30; 7.4%). Other indications included primary vasculitis (*n* = 11; 2.7%), mixed connective tissue disease (MCTD; *n* = 6; 1.5%), and autoimmune liver disease (ALD; *n* = 5; 1.2%). The umbilical cord was the main choice of source of MSCs in 385 cases (95.3%), and the second source of cells was the bone marrow in 17 cases (4.2%). Two patients received two different sources of cells in two separate infusions, respectively.

**Table 1:** Baseline characteristics of the study population

Variable	*n* (%)
Gender	
Female	344 (85.2%)
Male	60 (14.9%)
Age	
≤ 20 year	43 (10.6%)
20–40 year	179 (44.3%)
> 40 year	182 (45.1%)
Underlying disease	
SLE	178 (44.1%)
SS	72 (17.8%)
SSc	39 (9.7%)
RA	32 (7.9%)
PM/DM	30 (7.4%)
Primary vasculitis	11 (2.7%)
MCTD	6 (1.5%)
ALD	5 (1.2%)
Others	31 (7.7%)
The source of MSCs	
UC	385 (95.3%)
BM	17 (4.2%)
UC/BM	2 (0.5%)

*SLE* systemic lupus erythematosus, *SS* Sjögren’s syndrome, *SSc* systemic sclerosis, *PM/DM* polymyositis/dermatomyositis, *RA* rheumatoid arthritis, *MCTD* mixed connective tissue disease, *ALD* autoimmune liver disease

### Maintenance therapy for the underlying diseases

Before MSCI, all patients had received adequate but not effective treatment with conventional regimens including corticosteroids and immunosuppressants including cyclophospamide (CYC), methotrexate (MTX), mycophenolate mofetil (MMF), leflunomide (LEF), tacrolimus (FK506), tripterygium wilfordii Hook F (TWHF), and hydroxychloroquine (HCQ), for treatment of the underlying AID. The doses of corticosteroids and types of immunosuppressive regimens of patients when receiving MSC infusion were shown in Table 2. According to the procedure, all patients continued the treatment with steroids and immunosuppressants at the time of infusion. After MSCI, the rheumatologist can adjust the dosages or types of corticosteroids and immunosuppressants according to the patients’ condition. The patents in SLE group had higher prednisone doses compared with other disease group at the time of MSCI. After 5 years after the infusion, 90.1% of lupus patients had tapered their prednisone to 2.5–10 mg per day and 22.7% had discontinued immunosuppressants.

**Table 2:** Clinical characteristics of the AID patients enrolled in the study when receiving MSC infusion

Disease	Variable		*n* (%)
SLE	Disease course	≤ 12 m	27 (6.7%)
		12–60 m	75 (18.6%)
		> 60 m	76 (18.8%)
	Treatment course	≤ 12 m	52 (12.9%)
		12–60 m	77 (19.1%)
		> 60	49 (12.1%)
	Dose of steroids	≤ 10 mg	66 (16.3%)
		10–30 mg	102 (25.2%)
		> 30 mg	10 (2.5%)
	Types of immunosuppressants	0	32 (7.9%)
		1	56 (13.9%)
		≥ 2	90 (22.3%)
SS	Disease course	≤ 12 m	7 (1.7%)
		12–60 m	39 (9.7%)
		> 60 m	26 (6.4%)
	Treatment course	≤ 12 m	24 (5.9%)
		12–60 m	40 (9.9%)
		> 60	8 (2.0%)
	Dose of steroids	≤ 10 mg	51 (12.6%)
		10–30 mg	20 (5.0%)
		> 30 mg	1 (0.2%)
	Types of immunosuppressants	0	19 (4.7%)
		1	37 (9.2%)
		≥ 2	16 (4.0%)
SSc	Disease course	≤ 12 m	8 (2.0%)
		12–60 m	19 (4.7%)
		> 60 m	12 (3.0%)
	Treatment course	≤ 12 m	15 (3.7%)
		12–60 m	19 (4.7%)
		> 60 m	5 (1.2%)
	Dose of steroids	≤ 10 mg	26 (6.4%)
		> 10 mg	13 (3.2%)
	Types of immunosuppressants	0	3 (0.7%)
		1	23 (5.7%)
		≥ 2	13 (3.2%)
RA	Disease course	≤ 12 m	3 (0.7%)
		12-60 m	7 (1.7%)
		> 60 m	22 (5.4%)
	Treatment course	≤ 12 m	10 (2.5%)
		12–60 m	18 (4.5%)
		> 60	4 (1.0%)
	Dose of steroids	≤ 5 mg	15 (3.7%)
		5–10 mg	14 (3.5%)
		> 10 mg	3 (0.7%)
	Types of immunosuppressants	0	6 (1.5%)
		1	14 (3.5%)
		≥ 2	12 (3.0%)
PM/DM	Disease course	≤ 12 m	10 (2.5%)
		12–60 m	13 (3.2%)
		> 60 m	7 (1.7%)
	Treatment course	≤ 12 m	13 (3.2%)
		12–60 m	11 (2.7%)
		> 60	6 (1.5%)
	Dose of steroids	≤ 5 mg	11 (2.7%)
		5–30 mg	14 (3.5%)
		> 30 mg	5 (1.2%)
	Types of immunosuppressants	0	2 (0.5%)
		1	14 (3.5%)
		≥ 2	14 (3.5%)

*SLE* systemic lupus erythematosus, *SS* Sjögren’s syndrome, *SSc* systemic sclerosis, *RA* rheumatoid arthritis, *PM/DM* polymyositis/dermatomyositis, *m* month

### Hyperacute adverse events

A total of 48 (11.9%) patients had haAEs in 404 patients. Reactions included fever, headache, palpitation, facial redness, insomnia, and stomach discomfort. Palpitation and headache were the two most commonly reported haAEs during the infusion, both occurring in five (1.2%) of patients. Fever was the most commonly reported haAEs on D1 after infusion, occurring in 24 (5.9%) of patients. Eleven (2.7%) patients reported facial redness, and 11 reported insomnia at the first night after the infusion. Three patients complained of more night sweats than before infusion. Symptoms, such as stomach discomfort, petechiae at injection site, joint pain, and back pain, were reported in one (0.3%) of the patients. All these reactions were generally mild to moderate and classified as grade 1 or 2.

The patients with PM/DM or with age of > 40 years had higher incidence of haAEs compared with the relative groups (Table 3). The relations between underlying diseases or patient age and the incidence of haAEs were correlatively analyzed. The data showed that the underlying disease type and the patient age both had no effect on the incidence of haAEs (*p* > 0.05) (Table 3). The SLE group had the most haAEs, but statistical analysis showed that the incidence of haAEs did not correlate significantly with lupus course, treatment course, doses of corticosteroids, and types of immunosuppressive regimens.

**Table 3:** The associations of underlying diseases type and patient ages when they received infusion with the incidence of haAEs and total deaths, respectively

	haAEs (cases%)	Total deaths (cases%)
Diseases		
SLE	18 (10.11%)	14 (7.87%)
PM/DM	6 (20%)	11 (36.67%)
SS	9 (12.50%)	8 (11.11%)
SSc	7 (17.95%)	6 (15.38%)
RA	3 (9.28%)	3 (9.38%)
others	5 (9.43%)	3 (5.66%)
*p* value	> 0.1	< 0.05
Ages		
≤ 20 year	5 (11.63%)	4 (9.30%)
20–40 year	18 (10.06%)	9 (5.03%)
> 40 year	25 (13.74%)	32 (17.58%)
*p* value	> 0.1	< 0.05

*SLE* systemic lupus erythematosus, *PM/DM* polymyositis/dermatomyositis, *SS* Sjögren’s syndrome, *SSc* systemic sclerosis, *RA* rheumatoid arthritis

### Acute adverse events

A total of 16 patients had acute adverse events from D2 to D30 after infusion of MSCs. The incidence rate of aAEs was 4.0% (16 of 404) including one fever (0.3%), one hair loss (0.3%), and one peeling off of the skin (0.3%), two facial rash (0.3%), one cervical lymphadenopathy (0.3%), one leukorrhagia (0.3%), six infections (1.5%), two encephalorrhagia (0.5%), and one cirrhosis bleeding from esophageal varices (0.3%), causing five deaths. The first seven events were classified as grade 1 or 2. Six infections included four pulmonary infection, one herpes zoster infection, and one soft tissue infection, all leading to hospitalization admission and classified as SAEs. The total incidence rate of SAEs from D2 to D30 after MSCI was 2.2% (9 out of 404).

### Adverse events after 1 month of infusion

#### Organ system-related adverse events

##### Cardiovascular adverse events

Five patients had arrhythmias during infusion of MSCs, but no cardiovascular adverse events were reported after 1 month of infusion.

##### Gastrointestinal and renal adverse events

One patient reported stomach discomfort within the first day after MSCI, but no gastrointestinal adverse events were reported after 1 month of infusion. No renal adverse events were reported.

##### Pulmonary adverse events

No patients reported pulmonary adverse events after 1 month of infusion.

##### Neurological adverse events

Five patients had headache within the first day after infusion of MSCs, but no neurological dysfunctions were reported after 1 month of infusion.

##### Hematological adverse events

No patients reported hematological adverse events after 1 month of infusion.

#### Infection-related adverse events

**Fig. 1:**
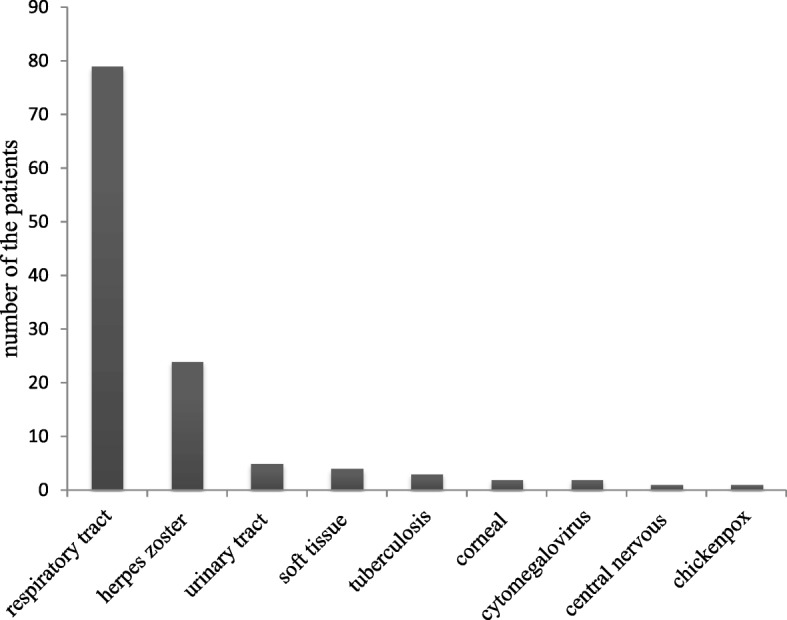
The frequency of infectious events in all patients post 1 month of MSCI. The numbers in the columns indicate the number of patients experienced infections

The frequencies of infection in 404 patients were depicted in Fig. 1. The incidence rate of total infections was 29.5% (119/404) and the rate of serious infections was 12.9% (52/404), in which 12 infections caused death. According to the site of infection, respiratory tract infection was the most frequent type of common infection, occurring in 79 patients (19.6%) in our cohort from the second month after MSCI, leading to 12 hospital administrations. Thirty-one patients (7.7%) reported a remarkably decreased incidence and/or duration of respiratory tract infection after MSCI compared with that before infusion, whereas three patients (0.7%) reported increased risk of respiratory infection. Acute urinary tract infection was documented in five patients (1.1%) and soft tissue infection was documented in four patients (0.9%). The mean TTI was 10.6 and 21.2 months in patients with acute urinary tract infection and soft tissue infection, respectively. In our study, two patients developed corneal infection, one patient suffered viral meningitis. Twenty-four events were classified as grade 3.

For opportunistic infections, herpes zoster infection was the most common one, occurring in 24 patients (5.9%). The median TTI was 29.9 months (range, 5 to 76). One patient had cytomegalovirus infection at 35 months post MSCI. Three patients developed lung tuberculosis, with a TTI of 15.3 months post-infusion. The frequencies of CMV infection and TB were 0.2% (1/404) and 0.7% (3/404), respectively. One patient developed chickenpox during the second month after MSCI. No cases of EB, hepatitis, or HIV were reported in this cohort. In the 24 patients with herpes zoster infection, 15 were lupus and 11 were >  40 year. The patients with SLE or with age of > 40 year had higher incidence of herpes zoster compared with the other groups.

#### Malignancy-related adverse events

During follow-up, five patients (1.2%) experienced malignancies with mean patient age of 53.6 years, including two SLE, one RA, one SS, and one SSc. The types of malignancies were two lung cancers, two colorectal cancers, and one bladder cancer. The mean TTC was 31.4 ± 19.55 months. Till the last follow-up, three patients had died of cancer.

#### Long-term adverse events: deaths

The data showed that deaths occurred in 45 patients. The median age of these patients was 46.1 ± 16.5 years, and the mean TID was 29.6 ± 23.4 months. The causes of deaths were infection in 12 patients (26.7%), disease relapse or complications associated with the underlying disease in 28 patients (62.2%), cancer in three patients (6.7%), and unknown causes in two patients (4.4%).

**Fig. 2:**
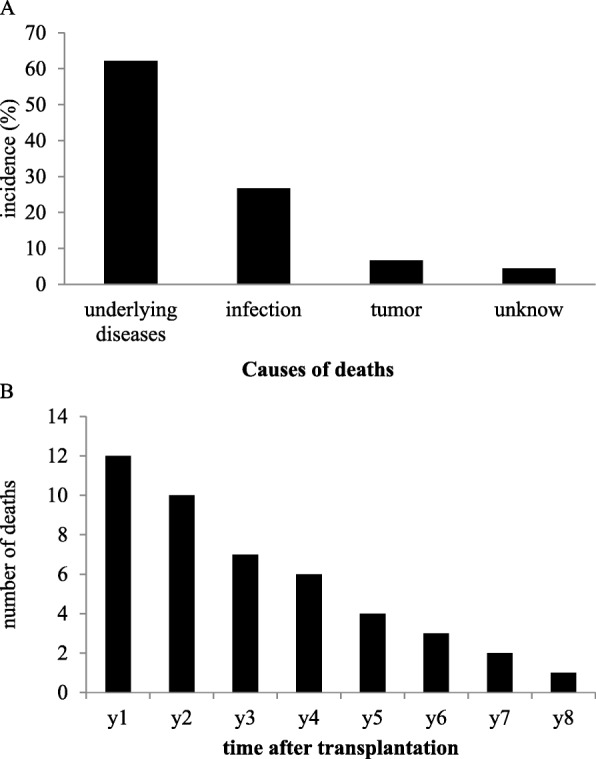
Cumulative deaths of 404 patients during the whole follow-up. The majority of deaths developed during the first 3 years after MSCI. year 1: the first year after MSCI; year 2: the second year after MSCI, and so forth. The numbers in each district represented the total deaths occurred in the different years

There were eight deaths within 100 days after infusion. Seven deaths were related with primary disease, and one was related with infection. So, TRM in MSCI in our study was very low with 0.2% (1/404). A very large part of deaths (29/45, 64.4%) developed during the first 3 years after MSCI (Fig. 2). Mortality 1 and 2-year in our patients was 3.0% (12/404) and 5.4% (22/404), respectively.

For the distribution of underlying diseases of 45 deaths, 14 (7.9%, 14/178) had SLE, 11 (33.3%, 11/30) had PM/DM, 8 (11.1%, 8/72) had SS, 6 (15.4, 6/39) had SSc, and 3 (9.4, 3/32) had RA. The SLE group had most death events (31.1%, 14/45) (Table 3), but PM/DM patients had the highest mortality compared with other diseases. Statistical analysis using Fisher’s exact test showed that death rates did not correlate significantly with disease course, treatment course, doses of corticosteroids, and types of immunosuppressive regimens in SLE and PM/DM group, respectively. In contrast, the type of underlying diseases was significantly associated with the death rate (*p* < 0.05) (Table 3).

The incidences of mortality on patient’s age ≤ 20 years vs. age 21–40 years vs. age > 40 years were 8.9% vs. 4.6% vs. 16.3%, respectively. Higher mortality was found in the patients with the age of more than 40 years old when receiving infusion of MSCs. The data also showed that the patients’ age was significantly associated with the death rate (*p* < 0.05) (Table 3).

### Survival rates

**Fig. 3:**
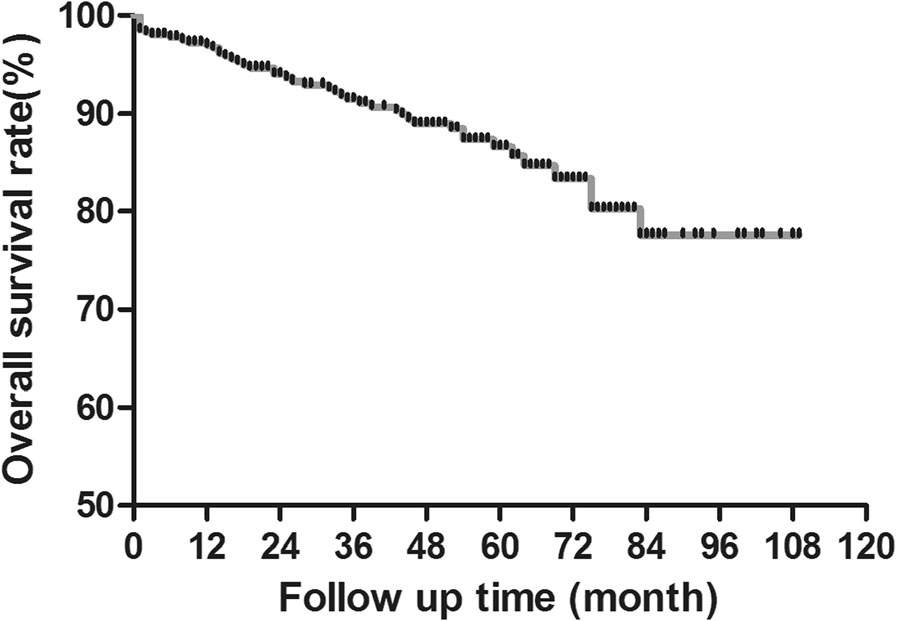
Overall survival rate of 404 patients within 9-year follow-up. The 5-year and 8-year survival rates 90.4% and 88.9%, respectively

At the end of the follow-up period, 359 patients (88.9%) were still alive with a median follow-up of 45.1 ± 25.7 months (range 2 to 109). Overall post-infusion survival rate was shown in Fig. 3. In the whole population, the 1-year and 3-year overall survival rates were 97.0% and 94.6%, respectively. The 5-year and 8-year survival rates were 90.4% and 88.9%, respectively.

**Fig. 4:**
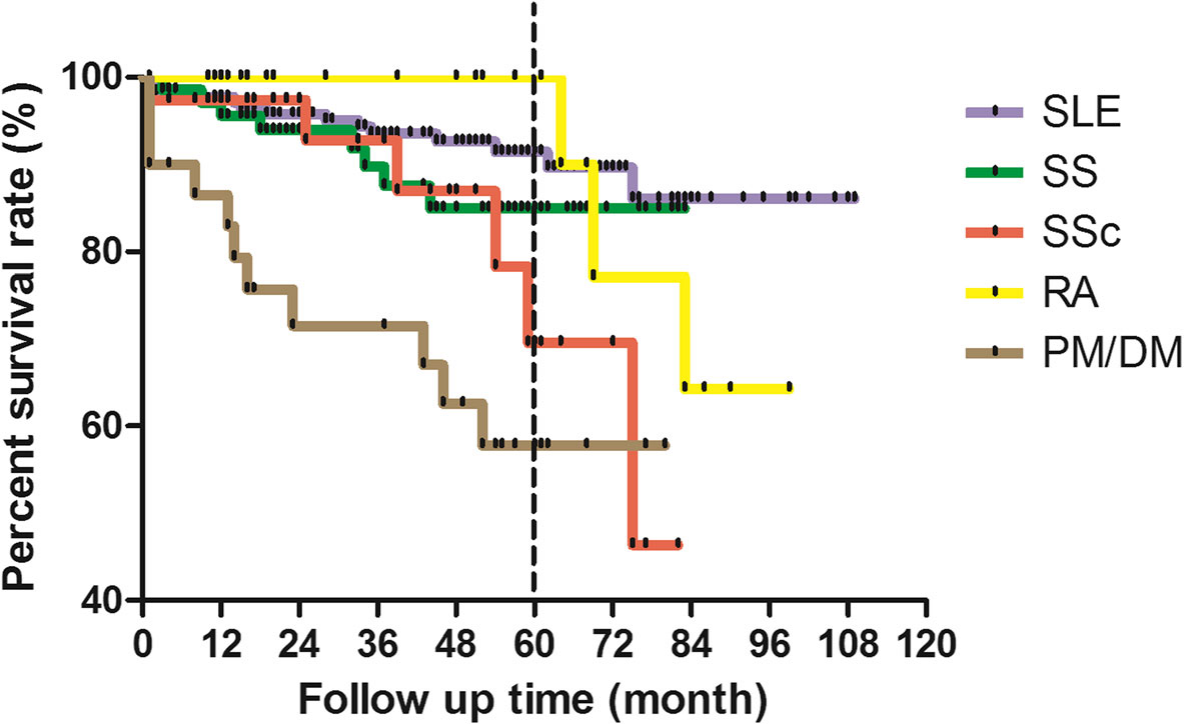
Survival rate of patients with different underlying diseases within 9-year follow-up. At 5-year follow up, survival rates for different diseases were ordered from highest priority to lowest as follows: RA, SLE, SS, SSc, and PM/DM

As shown in Fig. 4, survival rate of PM/DM patients was the lowest whereas that of RA patients was the highest within the first 6 years after infusion. At 5-year follow-up, survival rates for different diseases were ranked from high to low as follows: RA, SLE, SS, SSc, and PM/DM.

### Analysis in children

A total of 26 children under the age of 18 received MSCI, half with SLE. Two of them died of progression of underlying autoimmune diseases, both occurred after 100 days of infusion (19 and 45 months after MSCI, respectively). All other 24 patients are alive, in whom 8 have survived for 5 years and 16 have survived for 4 years. In these 26 patients, one reported insomnia during D1 after infusion and one had once herpes zoster infection during the follow-up period.

### Surgical operation

Surgical operations were classified as the noninfectious and nonmalignant events, and the frequency was evaluated. A total of 31 (7.67%) surgical operations were reported. Among these, 18 operations included four toe amputations, three liver transplantation, seven joint replacements, and four splenectomies, directly resulted from the primary disease. Other 15 operations had no direct connection with the underlying primary disease.

## Discussion

MSCs have been widely used due to their substantial multilineage differentiation and immunoregulating activities, in many fields ranging from autoimmune diseases, nervous system diseases, and orthopedic system diseases [18–21]. Many researchers have proved the satisfactory efficacy of MSCI in refractory AID patients. In our study, 32.5% patients in the SLE group after MSCI achieved major clinical remission and 27.5% patients achieved partial clinical remission at 12-month follow-up, whereas the 5-year disease remission rate in SLE group is 34% (data not shown). The remission rates in other groups have not yet been analyzed in view of the fewer cases. So far, few reports have emphatically focused on the adverse events after infusion. A comprehensive survey of adverse events will promote the clinical application of MSCI. So in this paper, we intend to characterize the occurrence of adverse events in 404 Chinese patients undergoing MSCI at one single medical center. In our cohort, about 64 AEs were reported during the first month after MSCI, in which AEs of grades 1–2 were more common.

Transplant-related mortality following allogeneic cell therapy, HSCT or MSCI, remains a major concern for patients and physicians. One of the most important findings of this retrospective observational study is that the TRM is lower in patients receiving MSCI compared with previous studies. Data from European Group for Blood and Marrow Transplantation (EBMT) registry and the Center for International Blood and Marrow Transplant Research (CIBMTR) registry had showed that the TRM in AID patients after autologous or allogeneic HSCT ranged from 5 to 12% [22–24]. In our study, the TRM is 0.2% (1/404). The high incidence of TRM in HSCT can be attributed mainly to the high toxicity of conditioning regimens. The conditioning regimen in HSCT consists of either total body irradiation or various combinations of chemotherapy including cyclophosphamide, busulfan, and melphalan each is toxic and increases the risk of mortality. Whereas MSCI, does not require conditioning regimens procedure and is well tolerated by patients.

The comparison of total death between MSCI and HSCT cannot be analyzed definitely because of the different follow up time in different studies. A recent retrospective study about autologous HSCT in diffuse cutaneous systemic sclerosis showed that the occurrence of death was 24.05% (19 out of 79 patients) during a median follow-up of 5.8 years [25]. For the whole AID population who received HSCT, the incidence of death was 111 of 900 patients (12.33%) between 1996 and 2007 in EBMT data and 49 of 368 patients (13.32%) between 1996 and 2009 in CIBMTR data [22, 23]. In our study, the occurrence of death was 11.1% (45/404) during 9-year follow-up. The reported frequent causes of death after HSCT were infections due to the toxic effects of drugs used during conditioning, AID progression, and organ failure. The most common cause in our study is disease relapse or complications associated with the underlying disease, which account for 62.2% of death causes. The difference was mainly relative to different infusion procedures, different stem cells and different proportion of disease patterns. However, undeniably, determinants of death also include severity of underlying disease, patient age, local medical level, family, and social ability to pay for treatment, family care, et al. Besides these, the original diagnosis appears to be the most relevant prognostic factor for mortality. Farge et al. reported there was a statistical difference of mortality depending on type of autoimmune disease, SLE and SSc patients had more deaths after HSCT [23]. Our data also showed that the type of underlying disease significantly associated with the death rate (*p* < 0.05). In our cohort, PM/DM patients had the higher mortality after MSCI compared with the patients with other AID. It was partly attributed to that many PM/DM patients selected in this study had rapidly progressive interstitial lung diseases, which itself was an important poor prognostic factor and a major cause of death [26]. The overall survival rate at 5 years in our study was 90.35%, appearing higher than that of AID patients receiving HSCT, with a 3-year survival rate of 86% in CIBMTR data and a 5-year survival rate of 85% in EMBT registry data [22, 23].

Infection and cancer are two important focuses in this study, which are also two critical concerns in the present field of MSCI. Until now, there are no any reports about the correlation between MSCI and the rate of infections and cancers. At baseline, patients with AID are more likely to develop infectious diseases and cancers than the general population in view of chronic inflammation and impaired immune system. Additionally, widely used medications including corticosteroids and immunosuppressants can raise the risks of infections and incidences of cancers [27]. A number of published articles have evaluated the risk of infections in AID patients [28–30]. In a Japanese hospital-based cohort study, there were 127 serious infections, including 43 intracellular infections leading to 8 deaths, in a total 604 AID patients during 2-year follow-up (127/1000 person-years) [28], which was not lower than that in this study. So, MSCI is not necessarily attributable to the increased incidence of infections and cancers of AID patients. Actually, MSCs have been shown to possess antimicrobial properties in several studies. MSCs can secrete antimicrobial peptides including the peptide LL-37, which can soften the bacterial cell wall and allow increased sensitivity to host and antibacterial agents [31]. The second cause of the important antimicrobial effectiveness of MSCs is to increase phagocytic activity of host immune cells in animal experiments [32–34]. The antimicrobial properties of MSCs is partly validated in this study that 31 patients (7.7%) reported a remarkably decreased incidence and/or duration of respiratory tract infection after MSCI compared that before infusion. The roles of MSCs in tumor cells growth are more complex. Whether the migrated MSCs have enhancing or promoting effects on tumor growth is not well defined, each theory has sufficient laboratory evidence to support. But to our knowledge, there were no clinical trials reporting the development of hematopoietic or solid tumors without exception, after the subjects received autologous or third party MSCs [35].

We cannot know whether there is any difference about the incidence rate of overall infections or cancers between AID patients receiving MSCI and HSCT, because we cannot find any data about that in AID patients receiving HSCT in published data while writing of this paper. We take CMV infection for an example, the reported prevalence in patients with different malignancies undergoing HSCT ranged from 16 to 65.5% in different studies [30, 36, 37], which is much higher than that in this study and suggested that MSCI is safer than HSCT.

Our study also has limitations. First, part of clinical data was about hospitalization in the local hospital, which was recounted by the patients or their families in follow-up or outpatient visits. Small differences in describing a symptom or a disease could lead to a different diagnosis of AE and may therefore lead to changes of outcomes. Second, the uncontrolled study design determines our data on AEs are mainly descriptive. And the uncontrolled assessments may have inflated effect sizes. More parallel controlled clinical trials are needed to be conducted to focus the safety of MSCI in the treatment of AID, which will expand the application of MSCI in clinical settings.

## Conclusion

Summing up, our article adds to the understanding of the occurrence of adverse events of AID patient receiving MSCI. The present study, drawing on data from a larger number of patients with longer follow-up, highlights new findings: whether TRM, or incidence of infection and malignancy are not high in AID patients undergoing MSCI; the occurrence of death after infusion are associated with type of underlying disease.

## Abbreviations

aAEs: Acute adverse events
AEs: Adverse events
AID: Autoimmune diseases
BM: Bone marrow
CYC: Cyclophospamide
haAEs: Hyperacute adverse events
HSCT: Hematopoietic stem cell transplantation
LEF: Leflunomide
MMF: Mycophenolate mofetil
MSCI: Mesenchymal stem cells infusion
MSCs: Mesenchymal stem cells
MTX: Methotrexate
PM/DM: Polymyositis and dermatomyositis
RA: Rheumatoid arthritis
SLE: Systemic lupus eryhtematosus
SS: Sjögren’s syndrome
SSc: Scleroderma
TNF-α: Tumor necrosis factor-α
TRM: Transplantation-related mortality
TTC: Time-to-cancer
TTD: Time-to-death
TTI: Time-to-infection
UC: Umbilical cord
